# Prevention and Treatment of Fungal Skin Infections Using Cationic Polymeric Films

**DOI:** 10.3390/pharmaceutics13081161

**Published:** 2021-07-28

**Authors:** Fritz Ka-Ho Ho, Albert Bolhuis, M. Begoña Delgado-Charro

**Affiliations:** Department of Pharmacy and Pharmacology, University of Bath, Claverton Down, Bath BA2 7AY, UK; K.Ho@bath.ac.uk (F.K.-H.H.); A.Bolhuis@bath.ac.uk (A.B.)

**Keywords:** dermatophytosis, fungal skin infection, *Trichophyton rubrum*, *Trichophyton interdigitale*, polymeric film

## Abstract

Dermatophytosis is a fungal infection of skin, nails and hair. Treatments can be long and infections are often recurrent, and novel treatments are desirable. Here we tested the use of polymeric films that can be sprayed on the skin for the prevention and treatment of dermatophytosis. The two polymers selected were ABIL T Quat 60 and Eudragit E100, which were tested ex vivo using a porcine skin model, and in vitro using microbiological and microscopy techniques. Acceptability of the polymeric films was tested on the skin of healthy volunteers. The results showed that ABIL and Eudragit films prevented and treated fungal skin infections. Whilst polymer films may provide a physical barrier that prevents fungal colonization, it was shown that both polymers are active antifungals ex vivo and in vitro and have intrinsic antifungal activity. For ABIL, we also established that this polymer binds essential nutrients such as metal ions and sugars, thereby restricting the growth of fungi. When applied to healthy subjects’ skin, the polymeric films neither modified the skin color nor increased trans-epidermal water loss, suggesting a low potential for skin irritation, and the approach was generally found to be acceptable for use by the volunteers. In conclusion, we developed a novel strategy for the potential prevention and treatment of dermatophytosis.

## 1. Introduction

Dermatophytes are fungi that mainly affect keratinised tissues such as skin, nails and hair. The infections caused by these fungi, referred to as dermatophytosis, are diseases with a high prevalence of about 20–25% of the world’s population [1]. Dermatophytosis is usually caused by fungi such as species of *Trichophyton*, *Microsporum* and *Epidermophyton*, all of which are fungi that invade and degrade keratin. The most common causative agents of infections in humans are *Trichophyton rubrum* and *Trichophyton interdigitale*, which in the UK comprise over 90% of all dermatophytes isolated [2,3].

Symptoms of the disease are usually fairly mild, but it does affect the quality of life of patients. However, complications can occur in some patients, such as in diabetics or those who are immunocompromised [4,5,6]. Treatment for these infections can be lengthy, and after treatment there is a high recurrence, with 25–40% of patients either relapsing or being re-infected [7]. There is also an increased incidence of failure of antifungal treatments [8,9,10], and novel prevention and treatment strategies are highly desirable.

Inspired by work on protective polymeric films for the coating of plant seeds [11,12,13], we envisaged a novel strategy to tackle fungal skin infections by using film-forming agents. These are typically polymers that can form a continuous thin layer on biotic or abiotic surfaces. They can act as active antimicrobial agents, or as excipients that increase the efficacy of antimicrobial agents by modifying and functionalizing monomers with active agents of functional groups. For instance, antimicrobial polymers have been used in a wide range of areas such as coatings that prevent spoilage of plant seeds, dental composites applications, food packaging and textiles [13,14,15,16]. Among others, cationic polymers may have the potential for providing activity against fungal skin infections. The antimicrobial properties of polymeric films have been documented, but less is known about their potential antifungal properties. One proposed mode of antimicrobial action is that cationic compounds bind to the negatively charged cell wall or membranes of microbes, which can increase their membrane permeability leading to cell lysis [17,18,19]. However, other modes of inhibition can be envisaged, such as polymers providing a physical barrier that prevents colonization [20]. Based on these modes of inhibition of antimicrobial polymers, we hypothesize that film-forming agents may provide a “drug-free” antifungal strategy that is less likely to lead to the emergence of resistance and that could provide a novel method of preventing and treating fungal skin infections. In this study, we focused on polymers already approved for cosmetic and pharmaceutical uses, which would facilitate clinical translation. Here, we tested two polymers, the first being ABIL^®^ T Quat 60 (hereafter referred to as ABIL), which contains silicone quaternium-22, a triquaternary polymethyl siloxane. ABIL is used as an ingredient in shampoos and conditioners for the protection of hair. The second polymer tested was Eudragit^®^ E100 (hereafter referred to as Eudragit), an FDA-approved co-polymer of dimethylaminoethyl methacrylate, butyl methacrylate, and methacrylic acid (50:25:25). Here we use a porcine skin model for fungal infection that we previously developed [21], to test the ability of these polymers to form topical films that prevent or treat fungal skin infections.

## 2. Materials and Methods

### 2.1. Chemical Reagents

All chemicals were purchased from Sigma-Aldrich (St. Louis, MO, USA) or Thermo Fisher Scientific (Loughborough, UK), unless specified otherwise.

Minimal salts agar (pH 5.4) contained 50 mM ammonium phosphate monobasic, 5.75 mM potassium phosphate dibasic, 3.4 mM potassium phosphate monobasic, and 1.5% agar (Agar no. 1; Oxoid, Hants, UK). Stock solutions of zinc chloride, iron chloride, and copper chloride at 500 mg L^−1^ were prepared in Milli-Q water by zinc chloride (anhydrous, 98+%; Alfa Aesar, Heysham, UK), iron (III) chloride (reagent grade, 97%), and copper (II) chloride (reagent grade), respectively.

### 2.2. Film-Forming Formulations and Components

ABIL T Quat 60 was kindly provided as a sample by Evonik Nutrition & Care GmbH (Essen, Germany), and formulated in solutions of different concentrations (5%, 10%, 15%, 20%, 25%, and 30%) in 95% ethanol (100% ethanol, VWR, Fontenay-sous-Bois, France, with 5% distilled water). Eudragit E100, in granules, was received as a sample from Evonik Röhm GmbH (Darmstadt, Germany). Film-forming formulations of the latter polymer were made by dissolving the Eudragit (5%, 10%, 15%, 20%, 25%, and 30%) and the plasticizer triethyl citrate (20% *w*/*w* of the dry polymer; ≥98.0%) in 95% ethanol [22,23]. The formulations were stirred for 24 h until clear solutions were formed.

### 2.3. Fungal Strains and Culture Conditions

The *Trichophyton* strains used in this study were *T. interdigitale* (ATCC 9533, human clinical isolate) and *T. rubrum* (ATCC 28188, human lesion isolate). Microconidia were isolated as described before [21].

Colony-forming units (CFU) of *Trichophyton* species were measured by serial dilutions and plate counting on Sabouraud dextrose agar (SDA).

### 2.4. Ex Vivo Antifungal Activity Tests

The potential prevention and antifungal activity of ABIL were studied by scoring the fungal growth on explanted porcine skin that was obtained as abattoir material and prepared and sterilised as described before [21]. Specimens of dermatomed skin pieces (~1 × 1 cm^2^) were placed on top of 25 mm polycarbonate membranes (CYCLPR PC BLK 25MM 0.2 µm; Whatman, Florham Park, NJ, USA) that were on minimal salts agar plates and left for 30 min before continuing with the experiments.

To assess the efficacy of the polymeric films in the prevention of dermatophytosis, the skin surface was treated with 40 µL of film-forming solutions containing different concentrations of either ABIL or Eudragit in 95% ethanol. As controls, 95% ethanol without polymer was used. The skin pieces were left for 2 h in a biological safety cabinet to maintain sterility, during which time the solvent evaporated completely. Then, 10 µL of conidia of *T. rubrum* or *T. interdigitale* (1 × 10^6^ CFU mL^−1^) was inoculated on the surface of the skin sections followed by incubation for 7 days at 30 °C.

To examine the potential use of the polymer films as a treatment for dermatophytosis, the skin sections were first inoculated with 10 µL of conidia of *T. rubrum* or *T. interdigitale* (1 × 10^6^ CFU mL^−1^), followed by incubation for 3 days at 30 °C until fully infected. After that, any mycelium on the skin surface was removed, and the skin sections were rinsed with sterile phosphate-buffered saline (PBS) twice, followed by washing with trypsin-EDTA (0.25%; Gibco, Paisley, UK). This process removes nearly all of the fungus from the surface, but fungal material remains in deeper layers of the skin [21]. Next, the samples were rinsed twice more with sterile PBS and transferred onto fresh minimal salts agar plates with a polycarbonate membrane placed between the skin and the agar and left for 30 min. Then, the top of the infected skin sections was treated with 40 µL film-forming formulations containing different concentrations of ABIL or Eudragit in 95% ethanol, or with 95% ethanol without polymer as control. The specimens were then incubated for 5 days at 30 °C.

After the incubation, images of the skin samples were taken and scored blind through an online questionnaire (Google Forms) by six volunteers external to the laboratory and not trained in microbiology. The volunteers scored the fungal growth from 0 to 5 based on their observation of mycelial coverage of the porcine skin surface, with 0 meaning no fungal growth, and a score of 5 referring to more than half of the skin piece covered by fungi (Table 1).

### 2.5. Scanning Electron Cryo-Microscopy

Using the prevention strategy as outlined in Section 2.4, skin specimens were first treated with ABIL and Eudragit, followed by inoculation with microconidia. These samples were then incubated for 24 h at 30 °C and visualized with scanning electron cryo-microscopy. For this, the skin specimens were directly mounted on SEM pin stubs with PELCO conductive silver paint (Ted Pella, Redding, CA, USA) and frozen immediately in liquid nitrogen. The specimens were then coated in a sputter coater and imaged by a Quanta 200 FEG SEM (FEI, Hillsboro, OR, USA).

### 2.6. In Vitro Fungal Growth Inhibitory Assays

The inhibition of fungal growth of *T. rubrum* and *T. interdigitale* by ABIL and Eudragit was examined on 96-well flat-bottom plates (Costar; Corning, NY, USA). The polymer films were prepared in the wells by adding 10 µL of film-forming solutions of ABIL and Eudragit, which were left overnight until dried out completely.

Fungal spores of *T. rubrum* or *T. interdigitale* were diluted in Sabouraud dextrose broth (SDB) at a concentration of 1 × 10^6^ CFU mL^−1^; 200 µL of these preparations was dispensed to each well. The plate was sealed with parafilm and incubated for 72 h on a microplate shaker (150 rpm) at 30 °C.

After the incubation period, the 96-well plates were analysed for viability using the phenol red assay (Section 2.7), or for the adherent biomass using crystal violet staining (Section 2.8).

### 2.7. Phenol Red Viability Assay

The fungal viability of *Trichophyton* species in the presence of ABIL and Eudragit films was performed using the phenol red viability assay [24]. Briefly, after the final incubation step described in Section 2.6, the medium in each well of the 96-well plate was transferred to a new well in a plate that contained 2 µL of 0.2% phenol red (indicator, ACS reagent grade) in DMSO in each well. After thorough mixing, the absorbance at a wavelength of 560 nm was determined using a microplate reader.

### 2.8. Crystal Violet Biofilm/Biomass Assay

The inhibitory effect of ABIL and Eudragit films on *Trichophyton* species biofilm formation was assessed by crystal violet staining as described [25] with some minor modifications. In brief, after the final incubation step described in Section 2.6, the medium with non-adherent cells was removed from each well. Then, the wells were washed twice with PBS and dried in an oven for 20 min at 60 °C. The fungal biofilm/biomass in each well was stained for 5 min with 150 µL of a 0.1% crystal violet solution. Next, the solution was removed, and the wells were immersed three times in a tray with cold tap water to remove excess stain. After that, 200 µL of 95% ethanol was added to each well to dissolve the crystal violet. After thorough mixing on a microplate shaker, the solution was transferred to a new 96-well flat-bottom plate, diluted if required, and read in a microplate reader at a wavelength of 595 nm.

### 2.9. Confocal Laser Scanning Microscopy (CLSM) In Vitro Study

*Trichophyton* spp. in the presence of different concentrations of ABIL were visualised by CLSM. Polymer films were formed by adding 40 µL of ABIL film-forming formulations or 95% ethanol (control) to each well of a Lab-Tek chamber slide (8 wells; Nunc, Rochester, NY, USA), followed by drying overnight. After that, 400 µL of *Trichophyton* spp. in SDB (1 × 10^6^ CFU mL^−1^) was dispensed in each well and incubated for 72 h, at 30 °C on a microplate shaker (100 rpm).

After incubation, 0.4 µL of SYTO 9 cell stain (10 mM; Invitrogen, Carlsbad, CA, USA) and 0.4 µL of NucFix Red (Biotium, Fremont, CA, USA) was added to each well, mixed thoroughly by pipetting gently up and down, and the samples were then incubated for 30 min at 30 °C in the dark. The solution and the slide chamber were subsequently removed and covered with a coverslip. The CLSM images were acquired as Z-stack 3D projections by Zeiss LSM880 with Airyscan laser confocal laser scanning microscope. The Z-stack images were displayed as 2D maximum intensity projection processed by ZEN 2.6 software.

### 2.10. Chelation of Zinc by ABIL

The ability of ABIL to chelate zinc was investigated by ^1^H NMR spectroscopic analysis. Zinc solutions (1–100 mg L^−1^) were prepared by addition of zinc chloride to methanol-D4 (D, 99.8% + 0.05% *v*/*v* TMS; Cambridge Isotope Laboratories, Inc., Andover, MA, USA) which also contained 20% of ABIL. The analysis was performed with a Bruker Avance III 500 MHz spectrometer with 16 scans recorded for each spectrum. The data were acquired and processed with Mnova 14.1. The ^1^H NMR prediction was processed by ChemDraw 18.0, targeting the shifting of the invariant proton at 2.22 and 2.32–2.36 ppm in all spectra.

### 2.11. Chelation of Iron by ABIL

The chelation effect of ABIL on ferric ions was studied by Inductively Coupled Plasma—Optical Emission Spectroscopy (ICP-OES). Polymer films were prepared by coating 30 mL universal tubes with 2.8 mL of 20% ABIL in 95% ethanol, which was left for 24 h on a tube rotator (55° fix-angle; 4 rpm) to enable film formation. Next, 7 mL of a 100 ppm ferric solution, prepared using iron (III) chloride (reagent grade, 97%), was added to each polymer-coated tube, and the samples left for 24 h on a rotator (55° fix-angle; 4 rpm). Then, the solutions were collected, and any polymer from these was removed using Amicon Ultra-15 centrifugal filter devices (10,000 MWCO; Merck Millipore, Cork, Ireland). The ferric ion concentration in the filtered solutions was measured by ICP-OES (Agilent 710 simultaneous spectrometer; Agilent Technologies, Santa Clara, CA, USA). The operational parameters are shown in Table 2. The Limit of Detection (LoD) and Limit of Quantification (LoQ) were determined from a calibration curve, calculated according to LoD = 3.3(σ/S) and LoQ = 10(σ/S), where σ is the standard deviation of the intercept, and S is the slope. The analytical and calibration parameters are shown in Supplementary Table S1.

### 2.12. Quadrupole Time-of-Flight LCMS (QTOF-LC/MS)

The potential interaction of carbohydrates with ABIL was studied by QTOF-LC/MS using glucose and mannose in a solution allowed to interact with the polymeric film. The ABIL film was formed by adding 80 µL of 20% ABIL in 95% ethanol to 7 mL bijou containers that were placed on a tube rotator (55° fix-angle; 4 rpm) for 24 h. Then, 2 mL of 100 µg mL^−1^ solutions of D-glucose (analytical reagent grade; Thermo Fisher Scientific) or D(+)-mannose (99+%, Acros Organics, Geel, Belgium) in Milli-Q water was added to the polymer-coated tubes and incubated for 24 h on the rotator (55° fix-angle; 4 rpm). After this time, the solutions were collected, and the polymer was removed using Amicon Ultra-2 centrifugal filter devices (10,000 MWCO; Merck Millipore, Cork, Ireland). The carbohydrate concentration of the filtrates was measured on an Agilent QTOF 6545 with Jetstream ESI spray source coupled to an Agilent 1260 Infinity II Quat pump HPLC with 1260 autosampler, and a column oven compartment (Agilent Technologies, Santa Clara, CA, USA). The system and data were controlled and analyzed via MassHunter Workstation software (B.07.00). The LoD and LoQ were determined as above, and the analytical and calibration parameters are shown in Supplementary Table S2.

An InfinityLab Poroshell 120 EC-C18 column (150 × 3.0 mm i.d., 2.7 µm particle size, 120 Å pore size; Agilent Technologies) was used and maintained at 50 °C. Isocratic elution with mobile phase A and B consisting of water (LC-MS grade, LiChrosolv; Merck, Nottingham, UK) supplemented with 0.1% formic acid (*v*/*v*, for mass spectrometry, 98%; Honeywell Fluka) and methanol (HiPerSolv; VWR) with 0.1% formic acid (*v*/*v*), respectively, using a diversion valve set to a loop capillary, a flow rate of 0.3 mL/min was used. A 5 µL injection volume was used.

The MS system was run using positive electrospray ionisation in multiple reaction monitoring mode. Nitrogen was used as the nebulising, curtain, and collision gas, with a flow rate of 12 L/min at 250 °C, while the nebuliser pressure was set at 45 psi. The sheath gas temperature and flow rate were set to 350 °C and 12 L/min, respectively. The Vcap, Fragmentor and Skimmer were set to 3500, 125 and 45, respectively.

### 2.13. In Vivo Study of the Acceptability of the Polymer Films in Healthy Subjects

This in vivo study was approved by the Research Ethics Approval Committee for Health at the University of Bath (EP 17/18 241). Seven healthy adult human volunteers (4 males and 3 females), who met the inclusion criteria (Table 3), gave written informed consent before participating in the study. The study aimed to assess the skin tolerability of polymeric films sprayed in vivo, their acceptability, and the residence time of the films on the skin. Before starting the experiment, both forearms were washed with tap water and dried with paper towels, after which application sites were demarcated by applying a frame of Mefix adhesive fabric dressing (Mölnlycke Health Care, Göteborg, Sweden) containing internal dimensions of 2.5 × 2.5 cm^2^ to the volar side of the arms.

For five (3 males, 2 females) of the seven volunteers, skin measurements were also performed. The skin colour was quantified non-invasively using a chromameter (CM-2600d, Konica Minolta, Osaka, Japan) and recording the a* value, which increases with skin redness that potentially could be due to irritation [26]. The transepidermal water loss (TEWL) was measured using an evaporimeter (AquaFlux AF 200, BioX, London, UK). A preliminary assessment of the residence of the polymers on the skin was by visual inspection and by the ease of removal of the film by washing with tap water at the end of the experiments. Skin color and TEWL baseline readings were taken at all sites at the beginning of the experiments. Film-forming formulations containing ABIL in 95% ethanol or control (95% ethanol without polymer) were applied to duplicate skin sites to the corresponding treatment area using an airbrush (AB9321; Sealey, Bury St Edmunds, UK) coupled to a mini airbrush compressor (AB900, Sealey), while film-forming formulations containing Eudragit in 95% ethanol and its control (95% ethanol without polymer) were applied to duplicate skin sites using a 100 µL pipettor. The latter was necessary as Eudragit formulations were too viscous to be applied with the airbrush used. The operational parameters of the airbrush are listed in Table 4. The values of skin redness and TEWL were measured at 15-, 45-, 75-, and 120-min post-treatment.

The a* variation (Δa*) or change in skin color was calculated by subtracting the baseline reading (a*_0_) from the value after the treatment (a*_treatment_) as described by Equation (1). The TEWL variation (Δ*TEWL*) was calculated as the ratio of post-treatment values (*TEWL_treatment_*) by the baseline reading (*TEWL*_0_) as described by Equation (2). Duplicate sites on the skin were used, and the mean values are reported.

(1) $$\Delta a^{*}=a_{treatment}^{*}-a_{0}^{*}$$

(2) $$\Delta TEWL=\frac{TEWL{\;}_{treatment}}{TEWL_{0}}$$

### 2.14. Data Analysis and Statistics

Results are presented as the mean ± standard deviation (SD), except for the results in the ex vivo antifungal activity tests, which are presented as median values. Statistically significant differences between two sets of data were determined by the Student’s *t*-test. For ordinal data, a Kruskal–Wallis analysis of variance (ANOVA) followed by the Dunn’s post hoc test was used for comparison of three or more data sets. A two-way repeated measures ANOVA was used for the in vivo acceptability study (Section 2.13). In all cases, the criteria for statistical significance were fixed at a *p*-value less than 0.05.

## 3. Results

### 3.1. Antifungal Activity of ABIL and Eudragit in an Ex Vivo Model

**Figure 1:**
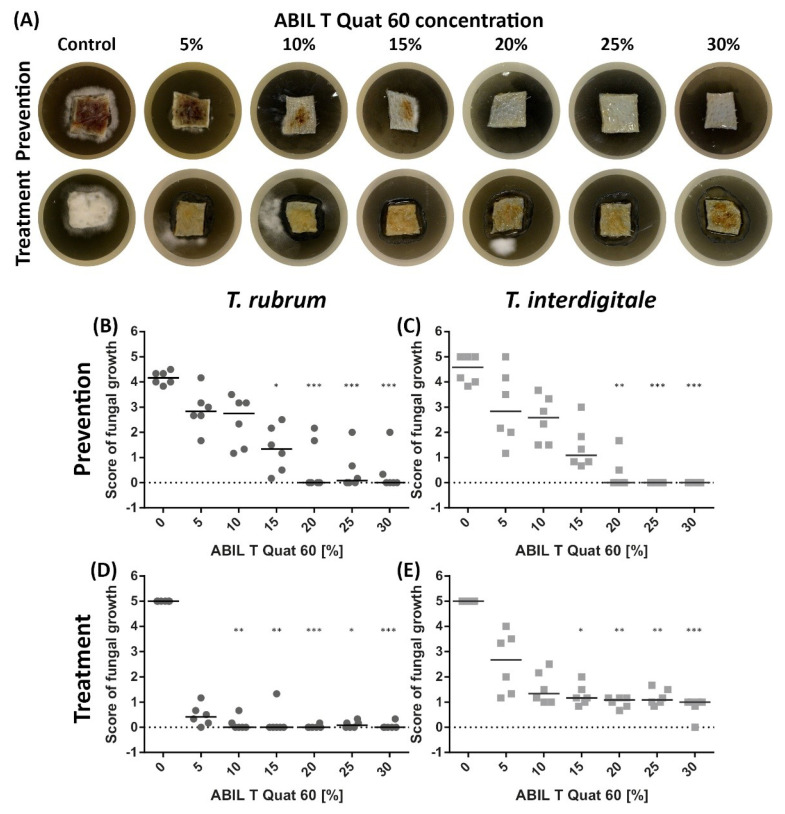
Effect of ABIL (5–30%) on the ex vivo fungal growth of *T. rubrum* and *T. interdigitale* in prevention and treatment experiments. (**A**) Representative images of fungal growth on porcine skin to which ABIL was applied before inoculation with *T. rubrum* (prevention) or treated with ABIL 3 days after inoculation with *T. rubrum* (treatment). Panels (**B**–**E**) show the median scores values for fungal growth (Table 1): (**B**): Prevention of *T. rubrum* growth; (**C**): Prevention of *T. interdigitale* growth; (**D**): Treatment of *T. rubrum* infected skin; (**E**): treatment of *T. interdigitale* infected skin. Statistical analysis was performed using a Kruskal-Wallis ANOVA followed by Dunn’s post hoc test: *n* = 6/group: * *p* < 0.05, ** *p* < 0.01, *** *p* < 0.001 vs. control.

**Figure 2:**
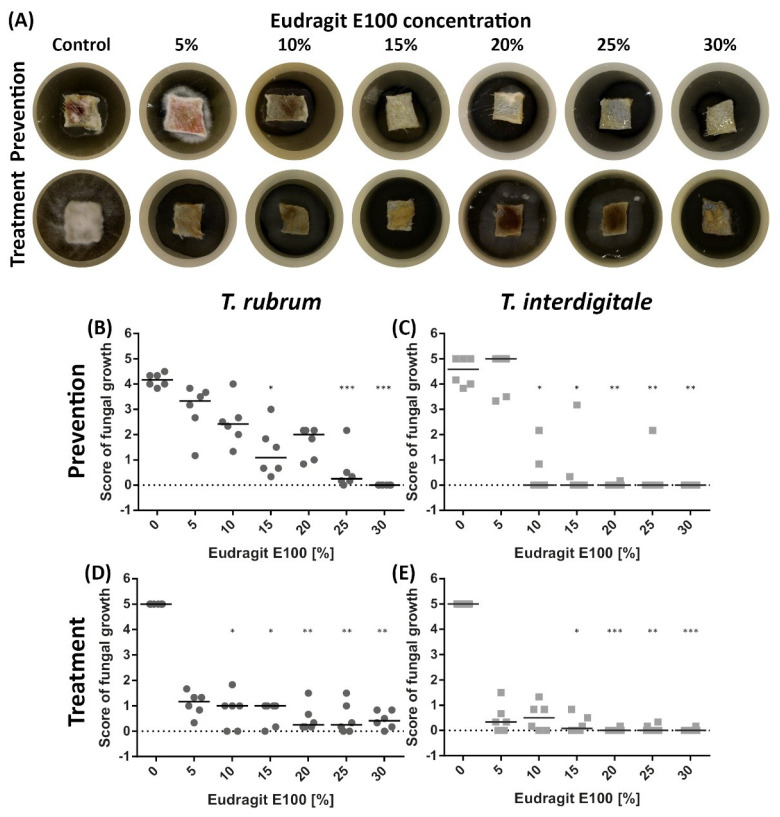
Effect of Eudragit (5–30%) on the ex vivo fungal growth of *T. rubrum* and *T. interdigitale* in prevention and treatment tests. (**A**) Representative images of fungal growth on porcine skin to which Eudragit was applied before inoculation with *T. rubrum* (prevention) or treated with Eudragit 3 days after inoculation with *T. rubrum* (treatment). Panels (**B**–**E**) show the median score values of fungal growth (Table 1): (**B**): Prevention of *T. rubrum* growth; (**C**): Prevention of *T. interdigitale* growth; (**D**): Treatment of *T. rubrum* infected skin; (**E**): Treatment of *T. interdigitale* infected skin. Statistical analysis was performed using a Kruskal-Wallis ANOVA followed by Dunn’s post hoc test: *n* = 6/group: * *p* < 0.05, ** *p* < 0.01, *** *p* < 0.001 vs. control.

Using our previously developed fungal skin infection model that employs ex vivo porcine skin [21], we analysed the ability of ABIL and Eudragit to prevent fungal infection on naive skin and to treat skin infected with *T. rubrum* or *T. interdigitale*. For this, a visual scoring test was used (on a scale 0–5), based on the percentage of skin covered by mycelial growth (Table 1). Representative images are shown in Figure 1A (ABIL) and Figure 2A (Eudragit), whereas Figure 1B–E and Figure 2B–E show the quantitative scores of the antifungal effects by the two polymers. Both the prevention and treatment potential were tested; in the case of prevention, the polymer was added before inoculation of the skin, whereas for treatment the polymer was added on skin that had been infected for 3 days. The model demonstrated the preventative activity of ABIL and Eudragit, with a clear relationship between the polymer concentration and a reduction in mycelium coverage with both *T. rubrum* and *T. interdigitale* (Figure 1B,C and Figure 2B,C). Similarly, the polymers were able to treat infected skin, with no or little recovery of fungal growth for 7 days after the treatment (Figure 1D,E and Figure 2D,E). In some of the treatment samples, mycelium could be seen growing around the edge of the polymeric film (see Figure 1A, 5%, 10% and 20%) without any visible growth on the treated skin. This suggests that some of the fungal material from the treated skin is still viable, but it does not lead to fungal growth on the treated skin. Curiously, treatment appeared to have a bigger impact on reducing fungal growth as compared to prevention (compare in Figure 1 and Figure 2; panels B with D, and C with E).

**Figure 3:**
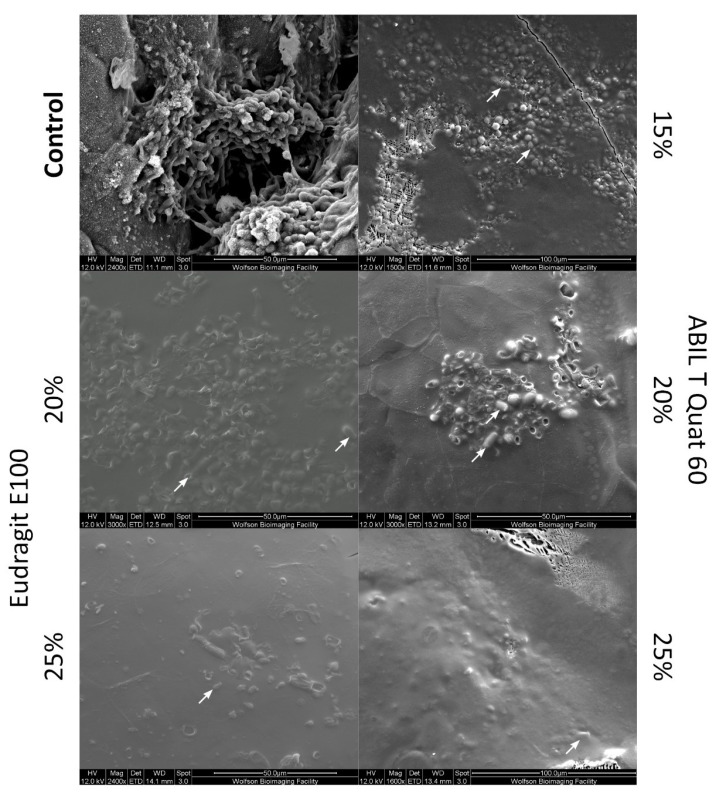
Cryo-SEM images of porcine skin pieces to which control solution (95% ethanol), or ethanol with ABIL (15–25%) or Eudragit (20–25%) were applied. This was followed by inoculation with *T. rubrum* conidia. After 24 h, conidia developing hyphae can be seen in the control, while a smooth surface was observed when polymers had been applied to the skin. The arrows indicate the positions of some of the conidia.

Cryo-SEM was used to visualize the effects of ABIL and Eudragit in the prevention strategy, in which polymer films were formed on porcine skin, followed by inoculation with microconidia. Based on the results of the ex vivo study (Figure 1 and Figure 2), cryo-SEM was used to study samples with polymeric films made with 15–25% ABIL and 20–25% Eudragit. In the absence of polymer, after 24 h of growth, conidia and developing hyphae are visible in the control images (Figure 3; control panel). When 15–25% ABIL was applied, only conidia were observed after 24 h, indicating that the polymer prevented germination of the spores. As the polymer concentrations were increased, conidia appeared completely embedded in the polymeric film. Similarly, the application of 20% or 25% Eudragit resulted in the conidia being embedded with polymer and failure of the conidia to germinate.

### 3.2. Antifungal Activity of ABIL and Eudragit In Vitro

**Figure 4:**
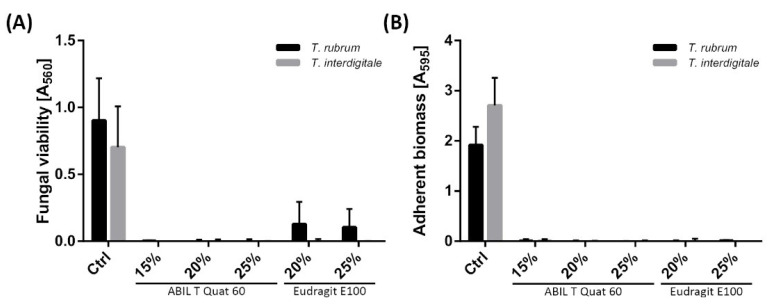
Inhibition of *T. rubrum* and *T. interdigitale* growth by ABIL and Eudragit based on fungal viability determined using phenol red (**A**) and adherence using crystal violet (**B**).

The antifungal activity of ABIL and Eudragit was tested in vitro by analysing fungal viability using a previously developed phenol red assay [24]. This was preferred instead of more common methods using the tetrazolium salt 2,3-bis-(2-methoxy-4-nitro-5-sulfophenyl)-2*H*-tetrazolium-5-carboxanilide (XTT), as the polymers interfered with the XTT assay (data not shown). With both polymers, the growth of *T. rubrum* and *T. interdigitale* was almost completely inhibited. Only with Eudragit, some growth was still observed with *T. rubrum*, but this was reduced approximately 8-fold when compared to the control (Figure 4A). Because of the residual viability of *T. rubrum*, we determined whether the fungi could form a biofilm by adhering to the plastic of the 96-well plates. This was done by washing out the non-adherent material and staining the remaining biomass with crystal violet, similar to a previously developed method for *Trichophyton* spp. [25]. As shown in Figure 4B, there was no adherent fungal biomass.

**Figure 5:**
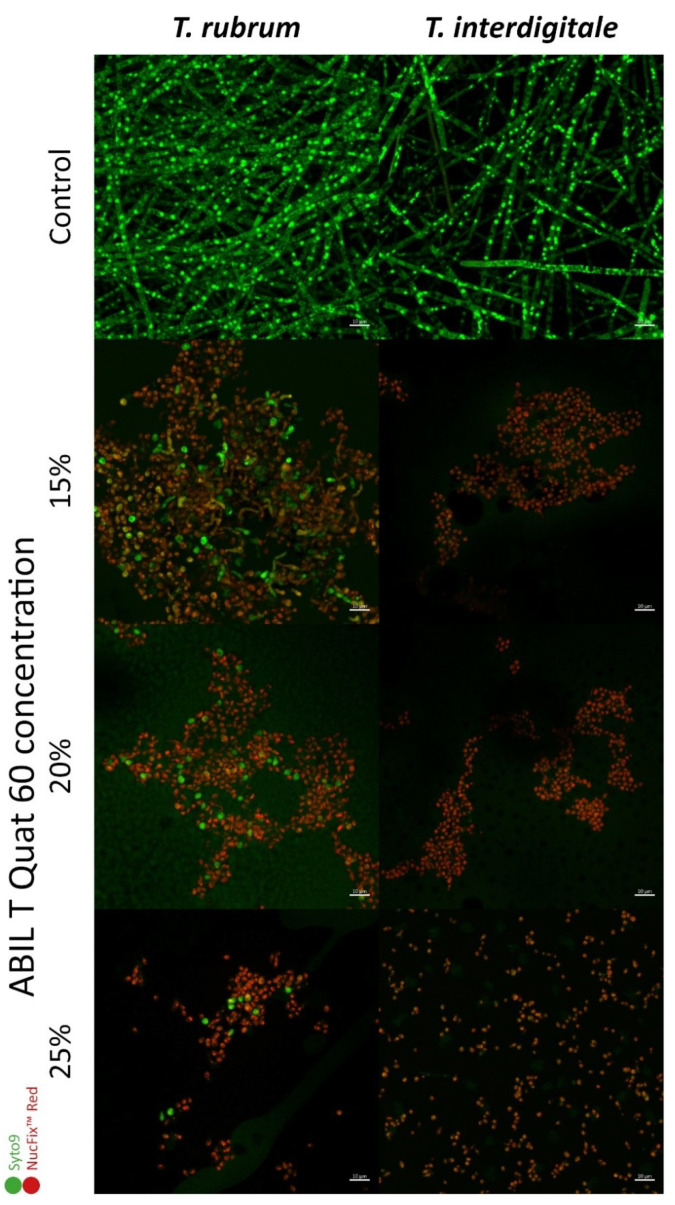
In vitro CLSM images of *T. rubrum* and *T. interdigitale* exposed to ABIL (15–25%) and stained with SYTO 9 (green = live) and NucFix™ Red (red = dead). Mycelium developed in the control of both *Trichophyton* spp., whereas the inoculated conidia did not germinate and were killed by the presence of ABIL.

To visualize the effect of the polymers, a Live/Dead stain was used on microconidia that were incubated for 72 h, using the DNA-binding dyes SYTO9 and NucFix Red to distinguish between living (green) and dead (red) cells. Unfortunately, this technique did not work with Eudragit due to the high autofluorescence and/or binding of the fluorescent dyes to the polymer, so only results for ABIL are shown (Figure 5). In the absence of polymer, both *Trichophyton* spp. developed into mycelium with multinucleated hyphae (in green), which is characteristic of filamentous molds. In contrast, in samples treated with ABIL, the red colour of NucFix Red dominated, and most fungal material still had the appearance of microconidia. This indicated that most conidia did not germinate, had damaged membranes, and were therefore probably dead. In the presence of 15% ABIL, some conidia with germ tubes could be seen with *T. rubrum*, but with 20% or 25% ABIL these germ tubes disappeared, even though some microconidia still appeared alive. With *T. interdigitale*, any level of ABIL tested resulted in the complete killing of microconidia, as demonstrated by the absence of any green staining.

### 3.3. Chelation Activity of ABIL

**Figure 6:**
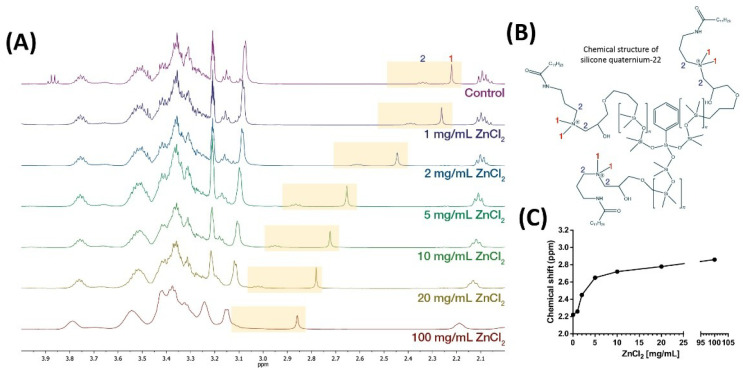
^1^H NMR titration study with 20% ABIL T Quat 60 and zinc chloride (1–100 mg/mL) in CD_3_OD. **1** and **2** represented the methyl group and the alkyl chain next to the amine group of ABIL. (**A**) Chemical shift induced by the chelation of zinc ion; (**B**) Chemical structure of ABIL; (**C**) Titration curve of the methyl group **(1)** of the amine group.

We noticed that the addition of ABIL (but not of Eudragit) to growth media resulted in a lighter colour of the growth medium, and therefore suspected that this polymer had the ability to bind to nutrients. We first investigated the polymer’s ability to chelate two metals, zinc and iron, that are important for several biological functions in fungi [27,28,29]. With increasing concentrations of Zn^2+^, NMR spectroscopy detected a signal shift indicating deshielding of the methyl group and alkyl chain protons next to the amine group of ABIL, denoted with 1 and 2 in Figure 6A (NMR spectra) and Figure 6B (chemical structure of ABIL). Based on the titration curve, the chemical shift increased with higher concentrations of ZnCl_2_ but did not change much further above 5 mg mL^−1^ (Figure 6C).

**Figure 7:**
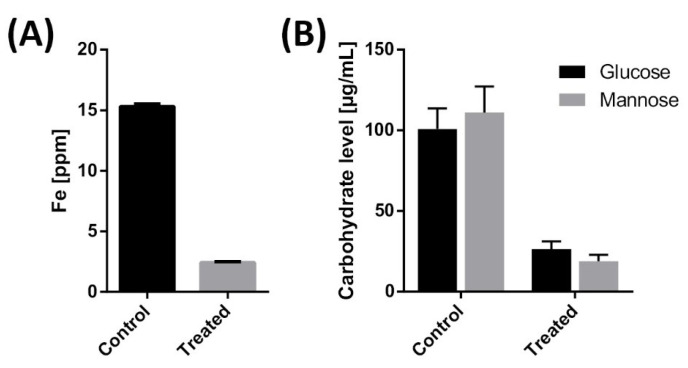
(**A**) Chelation activity of 20% ABIL T Quat 60 in ferric chloride solution measured by ICP-OES. (**B**) Reduction of carbohydrate level in the presence with 20% ABIL T Quat 60 measured by QTOF-LC/MS. Note that the initial concentration of ferric ion and both sugars decreased to values below the LoD in the treated samples (see Tables S1 and S2 in supplementary materials).

With ferric chloride (1–100 mg mL^−1^), similar effects as with ZnCl_2_ were seen, but due to the paramagnetism of the ferric ion, NMR signals were very broad, and no clear signals were detected at higher concentrations (data not shown). For this reason, the chelation of Fe^3+^ in tubes coated with an ABIL film was analysed using ICP-OES, which demonstrated that the concentration of ferric ions was reduced by 84% (Figure 7A).

Using QTOF-LC/MS, we also investigated the ability of ABIL to capture carbohydrates. In tubes coated with ABIL, the concentration of soluble glucose and mannose decreased approximately 4- to 6-fold (Figure 7B), but it should be noted that these values were below the statistical LoD as calculated from the calibration curve (see Supplementary data Table S2).

### 3.4. Acceptability of Film-Forming Polymers by Healthy Subjects

The results above support the potential application of these films to prevent and treat fungal skin infections, but further development of this approach requires that these agents do not have adverse effects on skin and are acceptable for patients. For this reason, we studied the effects of the polymeric films on human volunteers by measuring their potential effects on transepidermal water loss (TEWL) and skin redness using non-invasive probes, and their acceptability via a questionnaire that volunteers filled out based on their perceptions following topical application of the polymeric film-forming formulations.

**Figure 8:**
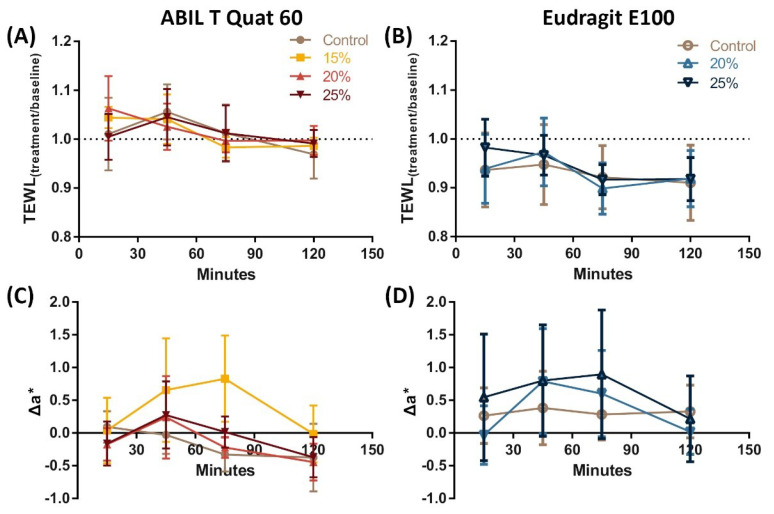
Evolution of TEWL (**A**,**B**) and skin redness (Δa*) (**C**,**D**) following application of polymeric film-forming systems to the forearm of healthy subjects: Films with ABIL (**A**,**C**) and films with Eudragit (**B**,**D**) (**A**–**D**: No significant differences found between treatments and control by Two-way RM ANOVA; subjects = 5).

**Figure 9:**
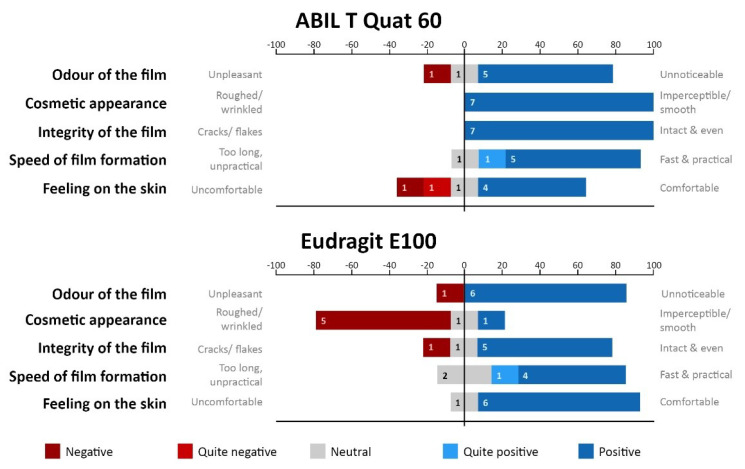
Acceptability of ABIL and Eudragit polymeric films as described by the participants’ (*n* = 7) answers to a questionnaire. The scoring system is based on a Likert scale.

First, we measured TEWL and skin redness before and after application of films formed from 15 (only ABIL), 20 or 25% polymer in 95% ethanol and of the control (95% ethanol) formulations to the volar side of forearms of five healthy volunteers. Post-treatment skin redness and TEWL were measured for up to 2 h post-application and, as shown in Figure 8 for both ABIL and Eudragit, there were no statistically significant differences between the values measured for skin sites treated with the solvent control and the polymeric films. Secondly, seven volunteers scored the acceptability of the films regarding their odour, cosmetic appearance, integrity of the film, speed of film formation, and feeling on the skin (Figure 9). The general perception of ABIL films was positive, with only one participant disliking the odour and feeling on the skin. As for Eudragit, the response was similarly positive with the exception of the cosmetic appearance, which 3 of the 5 volunteers disliked. In fact, Eudragit films had a somewhat wrinkled look on the skin, and one participant commented that the dried Eudragit film “appeared to pull on hairs in the skin”. Overall, there was a preference for ABIL films because of their softer and smoother feeling on the skin as compared to Eudragit. In all cases, the films remained in place for the length of the study. ABIL films seemed to be easily removed by rinsing with water, whereas Eudragit films were more “water-resistant” and persistent, and their removal required several repeats of washing with water and soap.

## 4. Discussion

In this study, cationic film-forming polymers were evaluated for their ability to prevent and treat fungal skin infections caused by the two of the most common dermatophytes, *T. rubrum* and *T. interdigitale* [2,3]. For this, we used a porcine ex vivo model previously developed by us [21]. This model fills a gap between in vitro studies and in vivo studies and, as demonstrated previously, is useful to study early stages of infection and treatment of dermatophytosis [21,24].

Our initial hypothesis was that film-forming polymers could provide a physical barrier between fungi and the skin and be useful for the prevention of dermatophytosis. Cationic polymers were first tested as the skin, including its outermost layer or stratum corneum, is negatively charged (isoelectric point 4.8) [30] so potentially it could interact with a film made from cationic polymers [31,32]. After an initial screening, two were chosen because of their promising results, which were ABIL and Eudragit. ABIL is a surfactant used as a conditioning agent for hair, whereas Eudragit E is an acrylate co-polymer that is used for pharmaceutical tablet coating, primarily to provide a protective coat and for taste-masking purposes [33]. The film-forming behavior by these two polymers was different, and whilst ABIL formed shiny smooth films when sprayed onto the skin, the Eudragit required addition of a plasticizer to provide some flexibility and, in the conditions tested here, could not be easily sprayed to form a film. The formulation of the polymers would thus require further work to improve their applicability.

Using the porcine skin model, we demonstrated that both polymers not only can prevent infection of the skin, but also can treat previously infected skin. This indicates that the polymers do not only function as a physical barrier that prevents fungal colonization but also have antifungal activity on their own. This provides an additional advantage as there would not be a need to add antifungal agents to the polymeric films. The antifungal activity increased as the films incorporated progressively higher concentrations of polymer in the solvent (Figure 1 and Figure 2), perhaps suggesting a better coverage of the skin by the polymeric film that provided better protection. In addition, conidia may be embedded in the polymer to a greater extent when the films incorporate a larger concentration of polymer, as could be observed with cryo-SEM with ABIL (Figure 3). The ex vivo results (Figure 1 and Figure 2) suggested the films to be more effective as treatments than as prevention tools, although this would need confirmation in vivo. One potential reason behind this observation is the longer, direct contact of ethanol with residual fungi in the skin. Whilst ethanol alone did not prevent growth as shown by the control experiments, its evaporation rate could have been reduced by the presence of the polymers, which could lead to longer contact time with the fungi. A second potential explanation could be that in treatment tests, the conidia and hyphae already present on the skin become coated with polymers, which leads to a greater antifungal effect as compared to prevention tests where microconidia are deposited on top of the film and thus are not fully coated with the polymer. Finally, it is also conceivable that because the stratum corneum and epidermis are already damaged in infected skin [21], the film-forming agents might access the infected epidermis to prevent further invasion of this layer. In the prevention model that uses intact skin, the polymers only cover the stratum corneum, and the microconidia are less likely to become entirely coated in polymer. In particular when using lower concentrations of polymer, it is also possible that the entire surface of the skin is not covered, creating some opportunities for conidia to develop and infect the skin. We have, however, not tested whether the polymer films form a thin homogenous layer on the skin, or whether they were heterogeneous with their thickness varying across different areas of the skin. Further experiments are also required to understand the differences between the prevention and treatment mechanisms of action, preferably using in vivo studies, and how to improve the efficiency of polymeric films, for example through manipulation of the film composition and properties.

In vitro, both polymers were also shown to be effective, as shown by the analysis of metabolic activity and adherence (Figure 4 and Figure 5). In the case of Eudragit, some viability was still observed with *T. rubrum*, and therefore we tested whether the fungi were able to form adherent biofilms that might be metabolically less active [25]. However, there was no adherence of *T. rubrum* in the presence of Eudragit, even though there was still some viable material present, indicating that one of the effects of Eudragit is to lower the ability of conidia or other fungal material to adhere to surfaces. Notably, we only tested this with Eudragit-coated 96-well plates, and whether adherence of conidia to the skin is also reduced in the presence of Eudragit will be addressed in a future study.

Using confocal microscopy (Figure 5), the antifungal effect of ABIL was confirmed through live-dead staining using the dyes SYTO9 (which is able to enter living cells) and NucFix Red (which is only able to enter cells with damaged cell membranes). Unfortunately, this technique was not successful for Eudragit, which binds to some fluorescent dyes, resulting in very high background fluorescence and making it very difficult to observe fungi. From the confocal images, it was clear that *Trichophyton* spp. were unable to develop a mycelium in presence of ABIL, with only *T. rubrum* forming some germ tubes and short hyphae at lower concentrations of the polymer. Interestingly, *T. interdigitale* was more sensitive to ABIL, as with this species no living material could be seen at any concentration of ABIL that was tested.

An important question is then the mechanism of action of these polymers. Previous studies showed that cationic polymers can bind to negatively charged cell walls and membrane of microbes, which leads to distortion of the cell envelope and an increase in cell permeability and lysis [17,19,34]. We also noticed that ABIL appeared to bind components in brown/yellow growth media that turned colourless when this polymer was added. This effect was not observed with Eudragit. Therefore, we investigated the ability of ABIL to bind to nutrients. Interestingly, this polymer was capable of chelating Zn^2+^ and Fe^3+^, presumably through interaction with an amine residue indicated by a shift in the NMR spectrum with increasing concentrations of Zn^2+^. The ability of chelators to inhibit fungal and bacterial growth by restricting the availability of essential ions has been demonstrated in many other studies [35,36,37,38,39]. ABIL was also able to bind glucose and mannose, and whilst the binding mechanism remains unknown it is important to note that the cell wall of fungi contains mannan, a polymer of mannose. Thus, whilst ABIL might prevent the growth of fungi by restricting access to nutrients, its potential interaction with mannan moieties on the cell envelope is also conceivable, providing another way for the polymer to kill fungi.

For Eudragit, the mode of action is less clear, but there are a few possible explanations. First, as it is a cationic polymer, it might bind to negatively charged cells walls of conidia or hyphae, thereby preventing germination and growth of the fungi. Secondly, it is possible that, as discussed above, Eudragit could reduce the binding of fungal material to the skin. Finally, as Eudragit forms a fairly hard and rigid film after drying, it may also be that this polymer creates a physical barrier, as our starting hypothesis proposed, preventing infection of the tissue underneath.

Despite the promising results described above, the use of polymeric films for this purpose requires the films to be well tolerated by the skin, i.e., they do not induce irritation or any other local side effects. The risks for local toxicity were greatly mitigated by selecting polymers already accepted as pharmaceutical and cosmetic ingredients. Nevertheless, as we were proposing a novel use that differs from their typical applications, we carried out a preliminary study on the potential ski irritation that could ensue after application. For that reason, we determined whether the polymeric films caused changes in TEWL and skin redness using non-invasive probes in healthy volunteers. Ideally, a film on the skin should not lead to a significant increase or decrease in water loss that could signal skin barrier damage and persistent occlusion, respectively. For instance, some penetration enhancers such as DMSO or terpenes increase TEWL as a result of their disruptive effect on the stratum corneum, whereas occlusive agents such as petrolatum reduce the breathability of skin [40,41,42]. Furthermore, any agent applied to the skin should not lead to a degree of skin redness that could signal skin irritation or vasodilation. Encouragingly, none of the participants showed signs of erythema or elevated TEWL at any of the multiple treated skin sites, as other studies with 6–7 volunteers had shown [43,44]. These preliminary data, consistent with the current pharmaceutical and cosmetic use of the polymers, supports the safety of these films and their translation to clinical use. Nevertheless, firm conclusions on acceptability require a significantly larger cohort and multiple applications. In addition, our study only involved subjects with healthy skin, and obviously, the safety of the polymeric films on skin infected with dermatophytes would require verification in clinical trials.

Finally, we evaluated the acceptability of the films by asking volunteers to fill out a questionnaire about the odour, cosmetic appearance, integrity of the film, speed of film formation, and feeling of the polymers on the skin. In general, feedback for both polymers was positive, except for the cosmetic appearance of Eudragit films that formed a fairly wrinkled and rigid layer on the skin. This could, however, be improved by using, for instance, other plasticizers or polymer mixtures that result in more flexible and pleasant-to-wear films. Whilst ABIL smooth and oily-like films were clearly more comfortable to wear, they were also very easily removed with washing. It follows that further formulation will need to balance wearing comfort, pleasant appearance, and satisfactory residence on the skin (as to avoid multiple applications). In regard to the latter, we did not do any measurements on polymers remaining on the skin after, for instance, washing. However, it was noticed by the volunteers that Eudragit was not easily removed by water, and some of the polymer film remained on the skin for extended periods of time (>24 h) and required several washing cycles to be removed completely. The residence of the very thin ABIL films was more difficult to assess, but overall they were not easily seen after a simple first wash. The difference in washability of the films clearly indicates that the frequency of application of the polymers needs to be investigated to provide ideal antifungal activity whilst considering convenience of use.

A final question to address is how the polymeric films could be used in practice. Currently used treatments for dermatophytosis are lengthy and often recurrent, with a risk of developing antifungal resistance [45,46], and novel non-antifungal treatments as those in this work present significant advantages. We envisage that these polymer formulations can be used as simple-to-apply sprays that would be available over the counter. They could be used for the prevention of dermatophytosis in people at higher risk of such infections. For instance, sports-practicing people could spray the skin of most exposed sites (i.e., feet) before entering changing rooms, where dermatophytosis is often picked up. Another example would be regular use by the elderly, who are also at increased risk, or other patients who often suffer from recurrent fungal skin infections.

## 5. Conclusions

In summary, both ABIL^®^ T Quat 60 and Eudragit^®^ E100 films provide a novel means of treating and preventing fungal skin infections. Importantly, both polymers have intrinsic antifungal activity, and therefore using them to prevent and treat dermatophytosis would not require the addition of antifungal agents. Both polymers are likely to have multiple modes of antifungal action that are rather generic, and it therefore seems unlikely that fungi would develop resistance against them. Preliminary data in healthy subjects suggests the films will be well-tolerated by the skin. Further work is required to completely elucidate the mechanisms of action by both polymers and optimize film properties.

## Figures and Tables

**Table 1:** Score criteria used by volunteers to assess blindly the fungal growth in skin samples. The score corresponds to the visually estimated percentage of the surface area of each skin piece covered by fungal growth.

Score	Visual Estimation of Fungal Coverage
0	No growth
1	<5%
2	<20%
3	<35%
4	<50%
5	>50%

**Table 2:** Operational parameters of ICP-OES.

Parameter	Value
RF generator	40 MHz
RF forward power	1300 W
Plasma gas (Ar) flow	15 L/min
Nebuliser gas (Ar)	0.9 L/min
Pump rate	7 rpm
Replicate readings	3
Studied wavelength (nm)	Fe: 238.204

**Table 3:** Inclusion criteria for participants.

Inclusion Criteria Met by All Participants
Healthy; aged between 18 and 72 years old
Lack of any skin disease
Male and female of any ethnic background
Willingness to provide basic information (i.e., age, gender, and health status)
Provide written informed consent before initiation of any study procedures
Agree not to participate in another trial during the study period
Able to communicate well with the investigators
Able and willing to adhere to the study restrictions and experiment schedule

**Table 4:** Operational parameters of airbrush.

Parameter	Value
Nozzle size	0.2 mm
Application angle	90°
Air pressure	40 psi
Application time	2 s
Application distance	10 cm

## Data Availability

All data are contained within the manuscript.

## Supplementary Materials

The following are available online at https://www.mdpi.com/article/10.3390/pharmaceutics13081161/s1, Table S1: Analytical and calibration parameters for the detection of iron using ICP-OES, Table S2: Analytical and calibration parameters for the detection of glucose and mannose using LCMS.
