# Supplementary material for: Prevention and Treatment of Fungal Skin Infections Using Cationic Polymeric Films
Source: Pharmaceutics. 2021 Jul 28;13(8):1161. doi: 10.3390/pharmaceutics13081161 (PMC8398677; doi:10.3390/pharmaceutics13081161)
Supplement: Supplementary file 1 [file pharmaceutics-13-01161-s001.zip › pharmaceutics-1280677-supplementary.pdf]

pharmaceutics

Supplementary Materials: Prevention and Treatment of Fungal Skin Infections Using Cationic Polymeric Films

Fritz Ka-Ho Ho, Albert Bolhuis and M. Begoña Delgado-Charro

Table S1. Analytical and calibration parameters for the detection of iron using ICP-OES.

Parameter	Fe
Calibration range	0.1–50 ppm
Limit of Detection (LoD)	3.57 ppm
Limit of Quantitation (LoQ)	11.92 ppm
R^2	0.99

Table S2. Analytical and calibration parameters for the detection of glucose and mannose using LCMS.

Parameter	Glucose	Mannose
Calibration range	10–200 μg mL^{-1}	10–200 μg mL^{-1}
Limit of Detection (LoD)	46.24 μg mL^{-1}	44.29 μg mL^{-1}
Limit of Quantitation (LoQ)	154.12 μg mL^{-1}	147.63 μg mL^{-1}
R^2	0.98	0.98
